# Evaluating context and interest in training in evidence-based mental health care: a qualitative investigation among healthcare providers in Kyiv, Ukraine

**DOI:** 10.1186/s13104-021-05786-3

**Published:** 2021-09-23

**Authors:** Kimberly Hook, Julia Kozishkurt, Olga Kovalchuk, Evelina Goncharenko, Vitalii Kodovbetskyi, Milana Opanasenko, Andrii Kopytko, Andriy Girnyk, Kateryna Kliuzko, Mari-Lynn Drainoni, Sergiy Bogdanov

**Affiliations:** 1 Department of Psychiatry, Boston Medical Center, Boston, MA USA; 2 Department of Psychiatry, Boston University School of Medicine, Boston, MA USA; 3 Department of Psychiatry, Massachusetts General Hospital, Boston, MA USA; 4 Center for Mental Health and Psychosocial Support, National University of Kyiv-Mohyla Academy, Kyiv, Ukraine; 5 Department of Medicine, Section of Infectious Diseases, Boston University School of Medicine, Boston, MA USA; 6 Department of Health Law Policy & Management, Boston University School of Public Health, Boston, MA USA; 7 Department of Medicine, Evans Center for Implementation and Improvement Sciences, Boston University School of Medicine, Boston, MA USA

**Keywords:** Global mental health, Psychotherapy, Training, Implementation science, Ukraine, Low- and middle-income countries

## Abstract

**Objective:**

Increasing access to quality, evidence-based mental health treatments, including psychotherapy, is a global priority. Knowledge of factors associated with delivery settings is critical to ensure that new practices are appropriate and effectively adapted for novel settings. Understanding perceived needs for training and interest in ongoing education is one key factor. This qualitative study aimed to identify perspectives on contemporary evidence-based psychotherapies, perceived needs for mental health training, and existing barriers and facilitators to provision of mental health services in community clinics in Ukraine. Purposive and snowball sampling was used to recruit 18 physicians and psychologists employed in community clinics in Kyiv. A combination of free-listing and semi-structured interviews was used to collect data, which were thematically coded using emergent coding.

**Results:**

Findings from this study indicated that participants recognize a need for improved mental health knowledge and training, as well as suggested interest and openness to learning short-term, structured psychological interventions. Additional barriers and existing strengths described by participants provide insight into possible factors that may impact future trainings in and implementation of modern mental health approaches.

## Introduction

More than 80% of mental health disorders occur among individuals living in low-and middle-income countries (LMICs), thus reflecting a disproportionally high burden of disease in often under-resourced settings [1, 2]. Despite evidence supporting the use of adapted evidence-based psychological interventions in commonly accessed health care settings, implementation of these practices into routine care has remained slow [3], which significantly contributes to the large gap in the availability of quality mental health services in LMICs [4, 5].

Few studies have evaluated mental health care provision in routine clinical settings in Eastern Europe [6]. In Ukraine (a LMIC [7] in Eastern Europe), it is estimated that 33% of the population experiences mental illness in their lifetime [8], though only 4.9% of such individuals receive treatment [9]. Accessing quality services (particularly psychosocial care) is also a challenge, partly due to psychologists commonly lacking formalized clinical skills training, including training in evidence-based interventions [8]. As part of significant mental healthcare reform, the Ukrainian Cabinet of Ministers passed the Mental Health Concept Note in 2017 endorsing improved access to evidence-based mental health care as a key priority [10]. While there are minimal investigations associated with evidence-based psychological therapies in Ukraine, an exception is a recent trial evaluating the adaptation and use of the Common Elements Treatment Approach (CETA), an evidence-based transdiagnostic psychological treatment intervention that targets mood, anxiety, and trauma symptoms [11]. As outcomes demonstrated that CETA effectively improved symptoms of depression, anxiety, and posttraumatic stress (*d* = 0.60–1.06), [12] there is current governmental and community stakeholder interest in scaling up and implementing CETA in routinely accessed clinical settings.

To gain preliminary knowledge about the delivery setting and gauge interest in future training efforts, this study aimed to identify perspectives on modern evidence-based psychotherapies, including CETA, and perceived needs for mental health training among current healthcare providers. We also evaluated existing barriers and facilitators in community clinics that affect mental health care provision and may have implications for future intervention adaptations.

## Main text

### Method

#### Setting and participants

We conducted a qualitative study with psychologists and physicians providing care in community clinics in Kyiv, Ukraine. We initially identified participants via shared institutional affiliations and by searching online listings of providers; we subsequently used snowball sampling to recruit additional respondents. Additional eligibility criteria included being 18 years of age or older and ability to complete the study in Ukrainian or Russian.

#### Data collection procedures

We followed data collection and analytic procedures outlined in the Design, Implementation, Monitoring, and Evaluation (DIME) manual, an approach specifically developed for rapid assessment in low resource environments [13]. This study was aligned with Module 1 of DIME, which offers guidelines for collection and analysis of qualitative data that will inform subsequent intervention adaptation and implementation. DIME methodology has been used worldwide, including previously in Ukraine [11, 14–16].

Data were collected from February 2020 through June 2020. Masters-level students and professors from the National University of Kyiv-Mohyla Academy conducted interviews, which occurred in-person or by telephone, were transcribed by hand, and were analyzed in the language of the interview. Interviews were conducted in Ukrainian or Russian by native, bilingual interviewers and followed an interview guide developed by the study team (see Table 1). We used a combination of free-listing [17] and semi-structured interviews, which allowed us to gather targeted data and also offered participants opportunities to elaborate on relevant topics.

**Table 1:** Interview questions

1	Please describe your current role.
2	Please describe the usual psychological services that patients are able to receive in your institution.
3	What problems do psychologists face in their daily work related to psychotherapy or psychological counseling?
4	Please describe the existing needs for further training and education among psychologists.
5	Imagine that the psychologists of this institution had the opportunity to be trained in a new method of psychological care which has been successfully tested in Ukraine. This is a short-term psychological counseling with about five to ten sessions, which can significantly reduce the symptoms of depression, anxiety and trauma. Please tell me, what comes to mind when you hear about this kind of psychological help? What are your thoughts, impressions, or previous experiences?
6	What expected barriers might occur during this training and intervention implementation?
7	What expected facilitators might occur during this training and intervention implementation?
8	How would the hospital or clinic administration respond to the training, education, and use of this type of intervention?
9	How do other health professionals (such as doctors) describe psychologists and their work?
10	Is there other information that you would like to share related to mental health services or mental health training needs?

This study was given an exempt IRB determination from our respective IRBs. Accordingly, we provided an informed consent template to all participants and obtained verbal consent prior to data collection.

#### Data analytic procedures

Following DIME’s approach [13], masters-level students participated in a day-long didactic and participatory training that built upon their prior research training. In order to ensure consistency and rigor in qualitative data collection and analysis, doctoral-level supervisors provided ongoing supervision. Pairs of interviewers met with each participant, and each interviewer recorded direct statements by hand (i.e., comments were not paraphrased or translated), thus resulting in two complete records of participant statements. Immediately after each interview was completed, the interviewing pair met together to review and reconcile their notes. For each completed interview, the interviewing pair conducted initial analysis by listing the different responses to each question and associating the interviewee identification number next to each response. When multiple interviewees provided similar responses, all interviewee identification numbers were listed next to that response [13]. In consultation with the rest of the analytic team, these responses were thematically coded using emergent coding. Final themes were agreed upon in consultation with fellow authors. Data were examined in ongoing discussions to allow for further understanding and to make connections between research questions and raw data.

### Results

Eighteen participants, each from a different clinic, were enrolled (see Table 2 for demographic characteristics), including ten physicians and eight psychologists. Below, we present main themes (see Table 3 for an overview of themes and descriptive quotes).

**Table 2:** Participant demographic characteristics

	Psychologists (*n* = 8*)	Physicians (*n* = 10)
Age (mean; range)	40; 28–49	42; 32–60
Gender		
Female *(n*)	6	5
Male (*n*)	1	5
Specialty areas (*n*)		
Cardiology	–	2
Dental medicine	–	1
Family medicine	–	1
Medical psychology	2	–
Neurology	–	1
Plastic surgery	–	1
Practical psychology	5	–
Psychiatry	–	3
Radiology	–	1

*One psychologist participant did not disclose demographical information

**Table 3:** Themes identified by physicians and psychologists

Themes	Descriptive quote
Physicians	
Perspectives on training	
Positive viewpoint	[I feel] positively [about training]. What surprises me is that someone doesn't want to know something.
Interest in time-limited treatments	I support short-term psychological counseling. It would be interesting to take a training course that helps you to manage the behavior of patients. The number of doctors interested in psychology is increasing. It would be interesting for doctors to listen and get such experience.
Identifying barriers	
Lack of referrals due to negative beliefs and stigma about mental health care	[There are] insufficient qualifications of psychologists. [There is] inconsistency of psychologists with the requirements for the treatment and diagnostic process.
Resistance from patients	The only barrier is that patients are often offended. They accepted it as an interference in their private life. The question is how to get it across to the patient tactfully. The patient comes to a cosmetic surgeon and is redirected to a psychologist; he [the patient] perceives it painfully.
Inadequate number of psychologists and limited time to see patients	[Psychologists are] short on time for the patient. There are many patients, but the psychologist is only one.
Logistical challenges: financing and lack of office space	For employees, training has to be free. If training is during working time, then it must be decided at the highest [administrative] level.
Identifying facilitators	
Recognition of the importance of mental health care and different specialist competencies	…there are competencies of a dentist and competencies of a psychotherapist.
Support from medical staff and clinic administration	The administration is ready to pay if it increases the hospital's income. If you [providers] do not have to pay—it is positive.
Psychologists	
Perspectives on training	
Need for training in evidence-based approaches	There are huge needs: in particular, the need for modern, evidence-based methods of psychotherapy. Our methods are copied from the West, without certification, and are outdated. Everything is outdated; everything needs to be changed to modern, evidence-based [practice]. I hear about a good effect from colleagues who practice cognitive behavioral therapy. I am lacking this knowledge.
Interest in time-limited, targeted treatments	[We need to] learn what to do when you cannot prescribe antidepressants and how to help a person with specific concerns. If it works, is effective, reduces symptoms, then I will be surprised. I would like to see this method in practice, and learn it. I have wanted [something like this approach] for a long time. I have an active desire for psychological structured counseling (rather than empty long hours of conversations about anything).
Identifying barriers	
Difficulty engaging patients in treatment	There is a great need to destigmatize the whole field of psychiatry in Ukraine. Social advertising is necessary, about [why] you need to work on mental health. We need information that it is normal to practice and be engaged in mental health care. Sometimes, it can be difficult to establish trust with patients, especially one who has a trauma [history]. If they suspect something, they will leave and never trust.
Unclear role of psychologists in clinic setting	But in our country, a psychologist is not equivalent to a psychiatrist. He is like a nurse, in contrast to the West, where a psychiatrist cannot make a diagnosis without a psychologist. Therefore, two-thirds of doctors do not understand the sense of the work of psychologists. They think that the psychologist is some kind of misunderstanding.
Role of psychologists is not valued	But there is some caste that is present. Even a medical psychologist—this is a person with less responsibility. We are an auxiliary part… a caste system is present in which the psychologist is inferior to the doctor. A medical psychologist has no career growth, but also no responsibility in comparison with doctors.
Concerns about time-limited interventions	Sometimes, short-term forms of treatment help. They work symptomatically. More often it doesn't help, because it's not only about removing symptoms.
Logistical challenges (interruptions, lack of office space)	In our hospital, there is no idea that you cannot go [in when] therapy is happening. Doctors themselves break these rules. Some of them are doctors from the old system who do not understand what psychotherapy is.
Ambivalent support from clinic administration	We have people who are more progressive than others, for example, our chief medical officer. But most people are suspicious of new methods. They believe that the Soviet methods work well, although there is no evidence of this. There are some administrations that are ambivalent: on the one hand, they understand the power of new methods, but on the other hand, they do not want to have unnecessary hassle, unnecessary checks of documents, etc.
Identifying facilitators	
Desire to improve patient outcomes	It is very motivating that I will be able to better help my patients, with whom I empathize. I want them to get better in any way.
Support for mental health providers on some treatment teams	Of course, people on the street (as I know) are not very serious about psychotherapy, [at least] most of them. But we do not have that in our work team. Everyone helps each other and understands the complexity of this area.
Opportunities to engage stakeholders	It would be a good idea to hold a conference first, to provide general informational support: what kind of methods, its results… And then conduct a seminar and supervision support.
Possible support from clinic administration	As far as we have heard, they are positive about this possibility, and they are ready to help. This is about [the general] hospital; for individual departments, it is unknown, although it is unlikely that anyone will put a spoke in the wheel.

#### Physicians: perspectives on training, identifying barriers, identifying facilitators

Overall, participants reported feeling positively about the possibility of increasing their knowledge of mental health care. Respondents indicated that they were not previously aware that targeted, time-limited psychological treatments exist and would be interested in learning more about these approaches.

However, physicians noted barriers that may challenge learning and implementing new mental health practices in routine care. First, difficulties associated with the referral process were commonly expressed; participants discussed general distrust of mental health care or negative beliefs about the qualifications and experience of psychologists. Accordingly, physicians reported less willingness to refer patients for mental health care. Further, several respondents noted encountering resistance from patients who are referred to mental health services, often due to patients’ limited understanding about the function of mental health treatment. Finally, participants described possible logistical challenges associated with implementing new psychological interventions, most frequently related to questions of financing and need for support from clinic administration. Other variables, such as lack of office space or lack of advertising regarding the availability of psychological services, were also noted.

In contrast, physicians identified a number of possible facilitators. Some participants endorsed the critical role of mental health care and discussed the utility of working with professionals from different specialty areas. Participants felt that both physicians and clinic administration would respond favorably to additional training in psychological treatments, particularly if positive results (either clinical or financial) came from their investment.

#### Psychologists: perspectives on training, identifying barriers, identifying facilitators

Participants broadly described existing needs for training in evidence-based practices, particularly due to knowledge gaps regarding modern psychotherapies. Psychologists consistently stated that their current methods are outdated and that the formal education they received did not adequately prepare them for clinical practice. However, participants endorsed general interest in training in a short-term psychotherapy and reported existing awareness of CETA. Respondents described interest in targeting and treating specific mental health concerns, as well as openness to using structured treatments. Participants stated that having existing documentation of CETA’s effectiveness, and understanding its limitations, would be useful in generating interest and uptake.

Psychologists reflected on challenges in their current work and possible barriers to integrating modern approaches. Several participants discussed difficulties with engaging patients in psychological care, often as patients either do not want or do not understand why they are recommended to start treatment. Stigma and distrust associated with mental health care are common patient barriers. Further, multiple participants noted that medical staff do not appear to understand the function of mental health care and the role that psychologists may play on multidisciplinary teams. In turn, many respondents reported feeling that medical professionals and clinic administration do not value mental health care and mental health staff. Stemming from these negative perceptions, many participants specifically referenced the lack of career opportunities and professional advancement in their clinical settings. Outside of these general barriers, some psychologists discussed concerns specific to learning and utilizing CETA (i.e., worries that a short-term treatment would only amount to symptom reduction in the short-term). Other logistical challenges, such as interruptions to clinical work and lack of office space, may affect psychotherapy treatment delivery. Finally, psychologists noted that clinic administration may be ambivalent about implementing a new treatment initiative (due to paperwork and other administrative hurdles) or may not allow psychologists to complete additional training during work hours.

Participants identified a number of facilitators that suggest openness and interest in learning and utilizing new methods. Importantly, many psychologists reported strong interest in learning a new approach, particularly if there is evidence that it will meaningfully impact patients. Psychologists also noted existing areas of professional support in their work settings, particularly among certain treatment teams that recognize the importance of mental health care. Participants felt that ensuring adequate supervision and support throughout implementation of new approaches would be mechanisms of increasing interest in training. Lastly, participants generally anticipated some level of support from clinic administration and noted that resistance from clinical staff would likely be minimal.

### Discussion

Across participants, interest in improving ability to address mental health symptoms, as well as a lack of training in mental health care, was almost uniformly acknowledged, which has implications both for the quality of care that is currently provided and for future professional development needs. Encouragingly, both psychologists and physicians expressed interest in learning more about mental health interventions, which may facilitate implementing a new approach into routine care. Specifically supporting the use of CETA, both groups of respondents predominantly expressed interest in learning about short-term, evidence-based psychotherapies. Nevertheless, both psychologists and physicians acknowledged a lack of knowledge and training about evidence-based psychotherapies, and to some extent, a lack of knowledge about mental illness. While Ukraine differs from other LMICs in that it has a large number of mental health providers [18, 19], respondents readily acknowledged that they are under-trained and thus may benefit from some of the same approaches used elsewhere (e.g., training in basic counseling skills) to bolster service delivery.

Participants identified a number of barriers that currently challenge mental health care provision. First, both physicians and psychologists overwhelmingly reported a lack of coordination and communication between mental health and medical providers, which negatively impacts multidisciplinary efforts and limits the number of referrals to psychologists. Future trainings may improve this concern by providing education to physicians about the role and training of mental health providers and by incorporating strategies that improve professional inter-group contact and communication patterns [20–22]. Some physicians also reported less inclination to work alongside or provide referrals to psychologists due to negative perceptions about their qualifications. Correspondingly, psychologists reported feeling disrespected or undervalued by both physicians and clinic administration. Implementation of psychotherapy training in community clinics may be a means through which to improve mental health training, increase visibility about provision of evidence-based psychotherapy, and improve working relationships between physicians and psychologists.

Finally, physicians and psychologists identified a number of challenges associated with mental health referrals. In part, lack of mental health knowledge appears to limit physicians’ ability to knowledgably discuss mental health care with patients. Other literature notes that patients may notice and internalize negative beliefs that physicians have about mental health referrals, which in turn limit patient engagement in mental health care; however, psychologists may aid this process by offering insights into how to more effectively communicate about mental health treatment [23]. Improving the quality of mental health services provided may also improve patient engagement. Lastly, participants noted that patients often lack understanding about mental health treatment and described significant levels of stigma associated with mental heath care [24, 25], which suggest that efforts to reducing stigma and increase mental health literacy among all stakeholders will be critical [26, 27].

### Future directions

This preliminary study highlights the current gaps in the provision of evidence-based psychosocial care in community clinics in Ukraine, as well as indicates interest and openness to ongoing training in contemporary approaches among healthcare providers. Future work should continue to focus on opportunities to build capacity and strengthen care provision in order to improve the accessibility and quality of mental health care services in low-resourced environments.

## Limitations

Several limitations should be noted regarding the implications of this research. First, we used a combination of purposive and snowball sampling to recruit participants, which may have biased our study results and been non-representative of the views of other healthcare providers (e.g., providers who chose to participate may have had stronger reactions or specific exposure to mental health care, as compared to other providers who may have had more neutral opinions). Similarly, our sampling strategy may have resulted in missing essential factors associated with implementation determinants. Follow-up individual interviews are needed to expand upon the initial ideas provided by the participants, and involving other stakeholders, including clinic administration and patients, would add nuance to the ideas presented in this paper. Further, while participants self-selected into our study, it is possible that power hierarchies between researchers and respondents may have impacted the viewpoints shared by providers. Finally, while participants offered ideas about anticipated barriers and facilitators, ideas presented in this paper are hypothetical and may differ in actuality.

## Data Availability

The dataset used and/or analyzed during the current study is available from the corresponding author on reasonable request.

## Abbreviations

CETA: Common Elements Treatment Approach
DIME: Design, Implementation, Monitoring, and Evaluation
LMICs: Low- and middle-income countries
